# Comparison of the Effect of Lidocaine Adding Dexketoprofen and Paracetamol in Intravenous Regional Anesthesia

**DOI:** 10.1155/2014/938108

**Published:** 2014-03-31

**Authors:** Ali Akdogan, Ahmet Eroglu

**Affiliations:** Anesthesiology and Intensive Care Medicine, Karadeniz Technical University, 61080 Trabzon, Turkey

## Abstract

*Objective.* Comparison of dexketoprofen and paracetamol added to the lidocaine in Regional Intravenous Anesthesia in terms of hemodynamic effects, motor and sensorial block onset times, intraoperative VAS values, and analgesia requirements. * Method.* The files of 73 patients between 18 and 65 years old in the ASA I-II risk group who underwent hand and forearm surgery were analyzed and 60 patients were included in the study. Patients were divided into 3 groups: Group D (*n* = 20), 3 mg/kg 2% lidocaine and 50 mg/2 mL dexketoprofen trometamol; Group P (*n* = 20), 3 mg/kg 2% lidocaine and 3 mg/kg paracetamol; Group K (*n* = 20), 3 mg/kg 2% lidocaine. Demographic data, motor and sensorial block times, heart rate, mean blood pressure, VAS values, and intraoperative and postoperative analgesia requirements were recorded. * Results.* Sensorial and motor block onset durations of Group K were significantly longer than other groups. Motor block termination duration was found to be significantly longer in Group D than in Group K. VAS values of Group K were found higher than other groups. There was no significant difference in VAS values between Group D and Group P. Analgesia requirement was found to be significantly more in Group K than in Group P. There was no significant difference between the groups in terms of heart rates and mean arterial pressures. * Conclusion.* We concluded that the addition of 3 mg/kg paracetamol and 50 mg dexketoprofen to lidocaine as adjuvant in Regional Intravenous Anesthesia applied for hand and/or forearm surgery created a significant difference clinically.

## 1. Introduction

Regional Intravenous Anesthesia (RIVA) was first applied by German surgeon, August K.G. Bier, in 1908, and this technique was defined as Bier block [1]. RIVA is generally preferred for patients who will have upper extremity surgery due to advantages such as providing a blood free surgery site, rapid onset and termination of the anesthetic effect, lack of necessity of severe sedation, and general anesthesia and easy application [2, 3]. Ketorolac, tenoxicam, paracetamol, clonidine, myorelaxant drugs, and opioids were added into local anesthetic agents as adjuvant to increase block quality in RIVA, to reduce tourniquet pain, to provide postoperative analgesia, and to reduce the dose of local anesthetic agent administrated [4–7].

Although molecular mechanism is not known well, intravenous paracetamol (perfalgan) is used for mild and intermediate postoperative pain. It is a nonopioid analgesic which reduces the opioid quantity used for severe pain [8–10].

Dexketoprofen trometamol is a nonselective NSAII with analgesic, antipyretic, and anti-inflammatory characteristics of which the parenteral form was developed in 2003 [11].

In the present study, we aimed to compare sensorial block onset and return periods, motor block onset and return periods, the block quality that appeared, preoperative and postoperative vital signs, and the need for intraoperative and postoperative analgesia for lidocaine-paracetamol combination and lidocaine-dexketoprofen combination retrospectively in the light of findings that we have obtained by the examination of patient files who have undergone hand and/or forearm surgery through the RIVA method in our university.

## 2. Material and Method

Records of adult patients referred to Karadeniz Technical University, Faculty of Medicine, Orthopedics Clinic and who have undergone hand and forearm surgery were enrolled. Once the study protocol was approved by the ethics committee of the Karadeniz Technical University in accordance with the 2nd Helsinki Declaration (date: 26.11.2012, meeting no.: 2012/125, resolution no.: 02), the anesthesia records of the patients were selected and the patients were enrolled in the study. Adult patients who have been examined routinely by anamnesis and physical examination and classified as ASA I and II according to preoperative physical status classification recommended by the American Society of Anesthetists were included in the study. Anesthesia records and hospital archive records of 73 patients between the age of 18 and 60 to whom regional intravenous anesthesia (RIVA) was applied were examined. The data of 13 patients were not included in the study because they did not comply with the study criteria, and the data of 60 patients were examined.

Exclusion criteria were (i) analgesic drug treatment in the previous 24 h, (ii) history of allergy to study medications, (iii) any neurological deficit in the upper extremities, and (iv) the presence of any contraindications to IVRA.

Age, gender, ASA, operation duration, and tourniquet periods were recorded from hospital archive files and anesthesia records.

It was observed from the files that premedication by 0.15 mg/kg midazolam (im) was performed before the surgery and RIVA (Regional Intravenous Anesthesia) was applied by monitoring average arterial pressure, heart rate, and peripheral oxygen saturation parameters.

The patients were divided into the following groups according to the medications used for RIVA procedure.

*Groups*

1. (Group D) lidocaine-dexketoprofen group: patients (*n* = 20) on whom RIVA was performed by the addition of 3 mg/kg 2% lidocaine and 50 mg/2 mL dexketoprofen trometamol (Arveles 50 mg/2 mL; UFSA Pharmaceuticals, Topkapı/Istanbul, Turkey) diluted with 0.9% normal saline to a total volume of 40 mL.
2. (Group P) lidocaine-paracetamol group: patients (*n* = 20) on whom RIVA was performed by the addition of 3 mg/kg 2% lidocaine and 3 mg/kg paracetamol (Perfalgan 1000 mg/100 mL vial, Bristol-Myers Squibb, France) diluted with 0.9% normal saline to a total volume of 40 mL.
3. (Group K) lidocaine-control group: patients (*n* = 20) on whom RIVA was performed by 3 mg/kg 2% lidocaine diluted with 0.9% normal saline to a total volume of 40 mL.

Records of these patients in three groups were examined.

It was observed that the tourniquet pressure of the RIVA solution was kept as 100 to 150 mmHg higher than systolic arterial pressure or at 250 to 300 mmHg, study medications were administrated within 90 seconds, sensorial block was assessed by a pinprick test every 30 seconds, sensorial examination of antebrachial, radial, ulnar, and median nerve dermatomes was conducted, and the motor block was assessed via the Modified Bromage Scale (MBS) by inability to move the wrist and fingers voluntarily by asking the patients if they could move their wrist and fingers. It was also observed that sensorial and motor block onset times and termination times of the blocks were recorded and their mean arterial pressures (MAP), heart rates, pulse oximeter, and oxygen saturations (spO2) were recorded and their records were evaluated.

It was detected that VAS (Visual Analog Scale) and Ramsey sedation scale were used before and at the 5th, 10th, 20th, and 30th minutes after tourniquet procedure and at the 5th, 10th, 15th, and 30th minutes and the 1st and 2nd hours after the tourniquet was opened for pain and sedation level measurements. Furthermore, intraoperative and postoperative analgesic requirements of the patients who had analgesic administration as fentanyl 1 *μ*g/kg when intraoperative VAS was over 3 were examined. It was observed that 500 mg oral Parol tablet was given to the patients whose pain sustained postoperatively and 50 mg contramal tablet for those whose pain was persistent. It was detected that interviews were performed with the patients after their discharge and questions related to operation comfort, quality, and incision pain were asked. Side effects that the patients had, such as nausea, vomiting, dyspeptic complaints, skin rash, and tinnitus, were examined from hospital archive files and anesthesia records.

Statistical data analysis was carried out by using “Statistical Package for Social Sciences” (SPSS) for Windows Release 13.0 program. Ki-Square was used for comparison of qualitative data; compliance to normal distribution in comparison of the data obtained by measurement was performed through the Kolmogorov-Smirnov test; student's *t*-test was used if it complied with the normal distribution and the Mann-Whitney *U*-test was used if it did not comply. Variance analysis of repetitive measurements or the Friedman test was used for comparison of measurements which continue from the beginning. Data obtained through measurements were expressed with mean standard deviation and data obtained by count was expressed as %. Significance level was accepted as *P* < 0.05.

## 3. Results

No difference was detected between the groups in terms of age, gender, ASA, operation durations, and tourniquet periods (Table 1).

No significant difference was found between the groups in terms of intraoperative and postoperative time values, heart rates, and mean arterial pressure values.

There was no statistically significant between-group type of surgery (Table 2).

Sensorial block onset durations of Group K were significantly longer than other groups (*P* < 0.05). There was no significant difference between Groups D and P in terms of sensorial block onset periods. No significant difference existed between the groups in terms of sensorial block termination times as well (Table 3).

Motor block onset durations of Group K were significantly longer than other groups (*P* < 0.05). There was no significant difference between Groups D and P in terms of sensorial block onset periods. Motor block termination duration was found significantly longer in Group D than Group K (*P* < 0.05) (Table 3).

**Figure 1:**
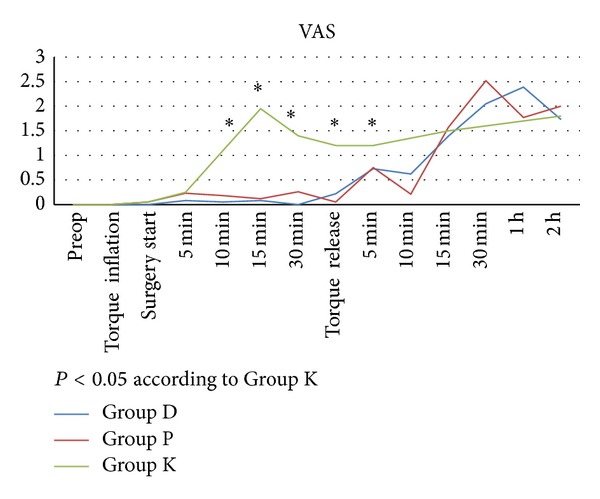
Intraoperative and postoperative visual analogue scale (VAS) scores (0–10).

VAS values of Group K were higher than other groups (*P* < 0.05). There was no significant difference in VAS values between Group D and Group P (Figure 1).

**Figure 2:**
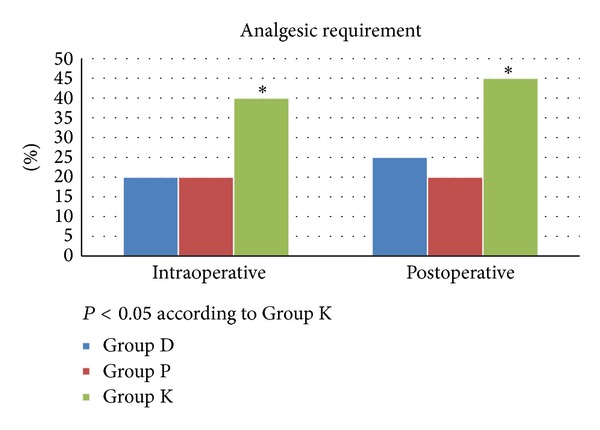
Analgesic requirement characteristics during the intraoperative and postoperative periods.

Intraoperative analgesia requirements were significantly more in Group K than Group P and Group D. Intraoperative analgesia was required for 8 patients in Group K and for 4 patients in Group D. Postoperative analgesia requirements were significantly more in Group K than Group P and Group D. Postoperative analgesia was required for 9 patients in Group K and for 5 patients in Group D (Figure 2).

It was also found that 1 patient had skin rash and 2 patients had bradycardia during their follow-ups. There was no significant difference between the groups (*P* > 0.05).

## 4. Discussion

Regional intravenous anesthesia is a common regional anesthesia method used for upper extremity surgery. It was detected that the addition of 3 mg/kg paracetamol and 50 mg dexketoprofen into local anesthetic agents as adjuvant in regional intravenous anesthesia performed for hand and/or forearm surgery reduced VAS values and shortened sensorial block onset time and motor block return time significantly when compared with patients to whom no adjuvant agent was added.

In RIVA, adverse events may appear as a result of local anesthetic agent passage into the circulation during intraoperative period and these complications may rarely be fatal. We attempted to reduce local anesthetic quantity and concentration and to find a local anesthetic with the lowest dose that may create an efficient local anesthesia to reduce systemic toxicity. Different adjuvant medications were added to local anesthetic agents to support a sufficient anesthesia on low concentration and dose.

In the literature scan, the most preferred local anesthetic agents for RIVA are prilocaine and lidocaine [12–14].

In the study conducted by Fahim et al., sensorial and motor block onset time was found to be shorter in the group where sufentanil was added to lidocaine; however, dizziness was detected following tourniquet opening [15].

Acalovschi et al. [16] and Fahim et al. added 100 mg tramadol into lidocaine [15], whereas Tan et al. [17] and Özcan et al. [18] added 50 mg tramadol appropriate and detected that the sensorial block onset time shortened.

There are studies indicating that the addition of dexamethasone might prolong sensorial and motor block in RIVA. Bigat and Boztuğ detected in their RIVA study conducted with dexamethasone, a steroid, by considering inflammatory steps during pain physiopathogenesis that 8 mg dexamethasone added to 3 mg/kg lidocaine increased anesthesia quality and provided a significant anesthesia on the first postoperative day [19].

Sen et al. concluded in their RIVA study by adding lornoxicam into 3 mg/kg lidocaine that sensorial and motor block onset time was shorter, sensorial and motor block return time was longer, and the necessity for first anesthesia for tourniquet pain was longer and total analgesic consumption was reduced in the group (L-IVRA) where lornoxicam was added to lidocaine when compared with other groups (control and L-IV) [20].

When the literature was examined, studies where paracetamol and dexketoprofen were added to local anesthetic agents in RIVA are rare [7, 21–23]. There is no study where two adjuvants were compared in the literature.

There is only one study where dexketoprofen was used as adjuvant in RIVA in the literature. Yurtlu et al. [23] detected in their study with dexketoprofen in lidocaine in RIVA that sensorial and motor block onset times were shorter, return times were longer, intraoperative analgesia requirement was less, intraoperative and postoperative VAS values were lower, and no difference existed between hemodynamic values. Similarly, motor block and sensorial block onset periods were found to be shorter in our study in patients to whom dexketoprofen was added when compared with the group without adjuvant addition. Furthermore, the need for intraoperative analgesia and VAS values were similarly found to be lower [23].

There are three studies in the literature where paracetamol was used as adjuvant in RIVA.

Ko et al. [7] reported in their RIVA study by adding 300 mg of intravenous paracetamol into 0.5% lidocaine that although sensorial block onset time was shorter in the group where paracetamol was added when compared with the control group, there was no difference in terms of sensorial block return times after the operation, intraoperative analgesia requirement was less, and intraoperative and postoperative VAS values were lower. Similarly, motor block and sensorial block onset periods were found to be shorter in our study in patients to whom paracetamol was added when compared with the group without adjuvant addition. Furthermore, the need for intraoperative analgesia and VAS values were similarly found to be lower.

In another study conducted by Celik et al. [22] through the addition of 200 mg of intravenous paracetamol to lidocaine (3 mg/kg), it was reported that there was no difference between sensorial and motor block onset and return times; furthermore, requirement of intraoperative analgesia was less. Similarly, we also found that the intraoperative analgesia requirement was less.

Sen et al. [21] did not find any difference between sensorial block onset time, motor block onset, and return time between the group where paracetamol was added and the control group in their RIVA study where they added 300 mg of intravenous paracetamol into lidocaine (3 mg/kg), and they reported that postoperative sensorial block return time was longer in the paracetamol group and intraoperative analgesia requirement was less.

When we assess the studies for possible adverse events and complications developed, no adverse events were detected in the study conducted by Ko et al. [7]. Sen et al. [21] reported nausea in three patients in their study. In case of follow-ups of our study, rash on one patient and bradycardia on two patients were detected. We could not find any significant difference between the side effects developed and groups.

There is no study in the literature in which motor and sensorial block periods, intraoperative analgesia requirement, hemodynamic monitoring, and side effects developed were compared to adding paracetamol and dexketoprofen to lidocaine in regional intravenous anesthesia. We analyzed anesthesia records and hospital records of the patients for whom 50 mg of dexketoprofen and 3 mg/kg paracetamol were added to 3 mg/kg in regional intravenous anesthesia.

According to our results, the addition of 50 mg dexketoprofen and 3 mg/kg paracetamol to 3 mg/kg lidocaine shortened sensorial and motor block onset periods and prolonged motor block and sensorial block termination periods when compared with the patients to whom adjuvant was not added in line with the studies conducted. Furthermore, it was found that it reduced intraoperative analgesia need and intraoperative and postoperative VAS values were lower; no significant difference existed in hemodynamic parameters. In the study conducted, no significant value was found when groups that adjuvant was added to were compared.

Consequently, it was found that the addition of paracetamol and dexketoprofen to the lidocaine in regional intravenous anesthesia applied for hand and/or forearm surgery does not create any significant difference; however, it is more successful clinically according to the group without adjuvant addition.

## Figures and Tables

**Table 1:** Demographic data, total operation time and total tourniquet, and application time (mean ± SD).

	Group K control (*n* = 20)	Group D dexketoprofen (*n* = 20)	Group P paracetamol (*n* = 20)
Age (year)	32.81 ± 12.15	35.65 ± 13.77	36.15 ± 13.55
Sex-male	13 (%65)	15 (%75)	15 (%75)
Sex-female	7 (%35)	5 (%25)	5 (%25)
ASA-1*	15 (%75)	16 (%80)	15 (%75)
ASA-2*	5 (%25)	4 (%20)	5 (%25)
Operation time (min)	39.0 ± 8.09	40.20 ± 9.29	48.55 ± 11.68
Tourniquet time (min)	54.3 ± 9.70	56.25 ± 10.25	66.15 ± 11.65

*ASA. American Society of Anesthesiologists physical classification status.

**Table 2:** Types of operations performed.

Type of surgery	Group K Control (*n* = 20)	Group D dexketoprofen (*n* = 20)	Group P paracetamol (*n* = 20)
Trigger finger	8	6	7
Carpal tunnel syndrome	5	4	6
Tendon release	4	7	5
Cyst excision	3	3	2

**Table 3:** Block onset times and block regression times of the groups (mean ± SD).

	Group K control (*n* = 20)	Group D dexketoprofen (*n* = 20)	Group P paracetamol (*n* = 20)
Sensory block onset time (min)	4.70 ± 1.38*	3.46 ± 1.14	4.6 ± 1.78
Sensory block regression time (min)	4.20 ± 1.73	4.1 ± 1.37	3.9 ± 1.99
Motor block onset time (min)	9.40 ± 4.23*	8.65 ± 2.97	10.05 ± 2.72
Motor block regression time (min)	4.70 ± 2.29	8.85 ± 1.72*	6.40 ± 3.18

*P < 0.05 according to Group D and Group P.
